# Changes in triglyceride-rich lipoprotein particle profiles in response to one-week on a low fat or Mediterranean diet by *TCF7L2* rs7903146 genotype: a randomized crossover dietary intervention trial

**DOI:** 10.1186/s12263-025-00763-y

**Published:** 2025-03-06

**Authors:** Chao-Qiang Lai, Julie E. Gervis, Laurence D. Parnell, Alice H. Lichtenstein, Jose M. Ordovas

**Affiliations:** 1 USDA ARS, Precision Nutrition Directive, JM-USDA Human Nutrition Research Center on Aging at Tufts University, Boston, MA USA; 2 Diet & Chronic Disease Prevention Directive, JM-USDA Human Nutrition Research Center on Aging at Tufts University, Boston, MA USA; 3 Precision Nutrition Directive, JM-USDA Human Nutrition Research Center on Aging at Tufts University, Boston, MA USA; 4 IMDEA Food Institute, CEI UAM + CSIC, Madrid, Spain; 5 CIBER Fisiopatologia Obesidad y Nutricion (CIBEROBN), Instituto de Salud Carlos III, Madrid, Spain

**Keywords:** *TCF7L2*, Mediterranean diet, Triglyceride-rich lipoprotein, Cardiovascular disease, Intervention trial

## Abstract

**Background:**

The *TCF7L2* gene is a significant genetic factor contributing to the risk of metabolic and cardiovascular diseases (CVD). We previously found that subjects with the TT genotype of *TCF7L2* rs7903146 variant, who consume a low-fat diet (LF) had a higher incidence of stroke than subjects with the CC genotype. Yet this association was abolished in subjects with the TT genotype who consumed a Mediterranean-type diet (MetD). However, the mechanism by which MetD diet modulates the association between *TCF7L2* and CVD risk is unclear. This study aims to validate these findings under real-world conditions and clinical practice to elucidate the biological mechanisms involved in this correlation.

**Methods:**

Thirty-five participants with BMI ranging from 27 to 34 kg/m^2^ were recruited based on rs7903146 genotype. Of those consented to participate, 21 had the CC and 14 had the TT genotype. Participants were randomly assigned to two dietary intervention groups, ensuring an equal distribution of CC and TT carriers. Each participant followed one of two diets (LF or MetD) for one week, followed by a 10-day washout period before switching to the other diet for one week. Blood samples were collected before and after each diet for metabolomic analysis using nuclear magnetic resonance (NMR) spectroscopy. The differential effect of the diets on triglyceride-rich lipoproteins was determined based on *TCF7L2* genotype.

**Results:**

The MetD significantly reduced triglyceride-rich lipoprotein concentrations compared to the LF diet. After consuming the LF diet, TT carriers exhibited more small VLDL particles, potentially contributing to CVD risk compared to CC carriers. However, this difference in risk was not observed with the MetD. Furthermore, the order in which the two diets were crossed affected the triglyceride-rich lipoprotein profile, with LF-MetD regimen showing a stronger effect on triglyceride-rich lipoproteins (TRL) levels than the MetD-LF regimen.

**Conclusions:**

Our findings suggest that rs7903146 TT carriers benefit more from a MetD than a LF diet in terms of their triglyceride-rich lipoprotein profile, which may reduce their risk of CVD. These results support the notion that genotype is a factor in determining the extent to which the MetD affects cardiovascular health.

**Supplementary Information:**

The online version contains supplementary material available at 10.1186/s12263-025-00763-y.

## Clinical Trials.gov

https://www.clinicaltrials.gov/study/NCT03458494

## Introduction

*Transcription factor 7-like 2* (*TCF7L2*) is the first genetic locus with common variants that were identified by genome-wide association studies to associate with type 2 diabetes (T2D) [1, 2]. This gene encodes a transcription factor that functions in the *WNT* signaling pathway [3]. In pancreatic beta-cells, TCF7L2 controls the production of GLP-1, a hormone that stimulates insulin secretion [3]. The genetic variant rs7903146 (C > T) in the *TCF7L2* gene, is associated with T2D [1, 2, 4]. Although the association has been demonstrated in several populations with different genetic origins, and was observed in one fifth of T2D cases [5], the exact mechanisms behind this association remain uncharacterized.

Our previous study in the PREDIMED trial examined the extent to which diet influences the impact of the *TCF7L2* rs7903146 variant on cardiovascular risk factors and CVD [6]. At baseline, individuals with the TT genotype exhibited a greater risk for T2D compared to those with the CC genotype [7]. Furthermore, adherence to a Mediterranean Diet (MetD) mitigated the adverse effects of the TT genotype on fasting glucose levels and lipid profiles. Essentially, higher compliance with the MetD counteracted the negative implication of the TT genotype. Moreover, after a follow-up period of approximately five years, TT individuals adhering to a low-fat (LF) diet displayed an increased incidence of stroke compared to CC individuals. However, this risk was almost fully negated among TT individuals who maintained a MetD [6]. Although the PREDIMED study provided strong evidence that MetD adherence can reduce cardiovascular risk by 30%, comparable to pharmaceutical intervention, this is derived from a primary prevention study [6, 8, 9], and does not explain the mechanisms behind the protective effects.

Postprandial lipemia (PPL) describes the alteration of plasma lipoproteins following a meal [10]. More than four decades ago, it was hypothesized that the post-meal rise in triglyceride-rich lipoproteins (TRLs) and their remnants contribute to atherosclerosis [10]. Consistently, follow-up research has shown a correlation between fasting levels of remnant lipoprotein cholesterol and triglycerides with the accumulation of TRLs after eating [11–13]. Very low-density lipoproteins (VLDL), a crucial TRL type secreted by liver, are composed mainly of triglycerides and cholesteryl esters, and are uniquely transporting apolipoprotein B [14, 15]. VLDL serves as a precursor to intermediate-density lipoprotein (IDL), which in turn becomes low-density lipoprotein (LDL), distributing cholesterol and triglycerides vital for numerous biological processes [14–16]. Beyond its physiological function, VLDL is implicated in cardiometabolic disease [16–21]. Larger VLDL particles from the fasting state, classified by size through nuclear magnetic resonance (NMR) spectroscopy, are linked to atherosclerosis and the prevalence of insulin resistance and diabetes [16, 18, 22]. A previous study showed that participants with TT genotype at *TCF7L2* rs7903146 exhibited an altered postprandial response in TG and triglyceride-rich lipoprotein (TRL) when they consumed a high-fat meal [23]. Our previous study found that *TCF7L2* genotype interacted with polyunsaturated fatty acid (PUFA) intake associated with fasting TG and VLDL, as well as postprandial responses in TG and VLDL [24]. TRLs directly contribute to intimal cholesterol deposition and foam cell formation, as well as plaque formation and progression [25]. TRLs are also involved in the activation and enhancement of several pro-inflammatory, pro-apoptotic and pro-coagulant pathways that are pivotal in the pathogenesis of atherosclerosis [26]. However, it remains unclear how different dietary patterns, especially those varying in fat quantity and type such as a LF diet compared to a MetD, might influence the distribution of VLDL (TRLs) with impact on cardiometabolic diseases.

Previous studies demonstrated that short-term dietary intervention mainly affects TRL concentration [27–29]. Hence, we focused on TRLs as the outcomes in our short-term dietary intervention trial. Given that *TCF7L2* variants are associated with the risk of cardiometabolic diseases [2, 5, 6], we hypothesized that carriers of different *TCF7L2* genotypes respond differently in TRLs to LF and MetD diets. Hence, the primary aim of this study is to examine the changes in triglyceride concentrations within TRL particles (notably, VLDL) after adhering to a LF or MetD for one week. Additionally, we seek to determine if these changes are affected by the *TCF7L2* rs7903146 genotype, specifically between the CC and TT genotypes. This investigation could provide important insights into the relationship between diet, genetic factors, and lipid metabolism, potentially informing dietary recommendations and personalized nutrition strategies.

## Study population and methods

### Study design

**Fig. 1:**
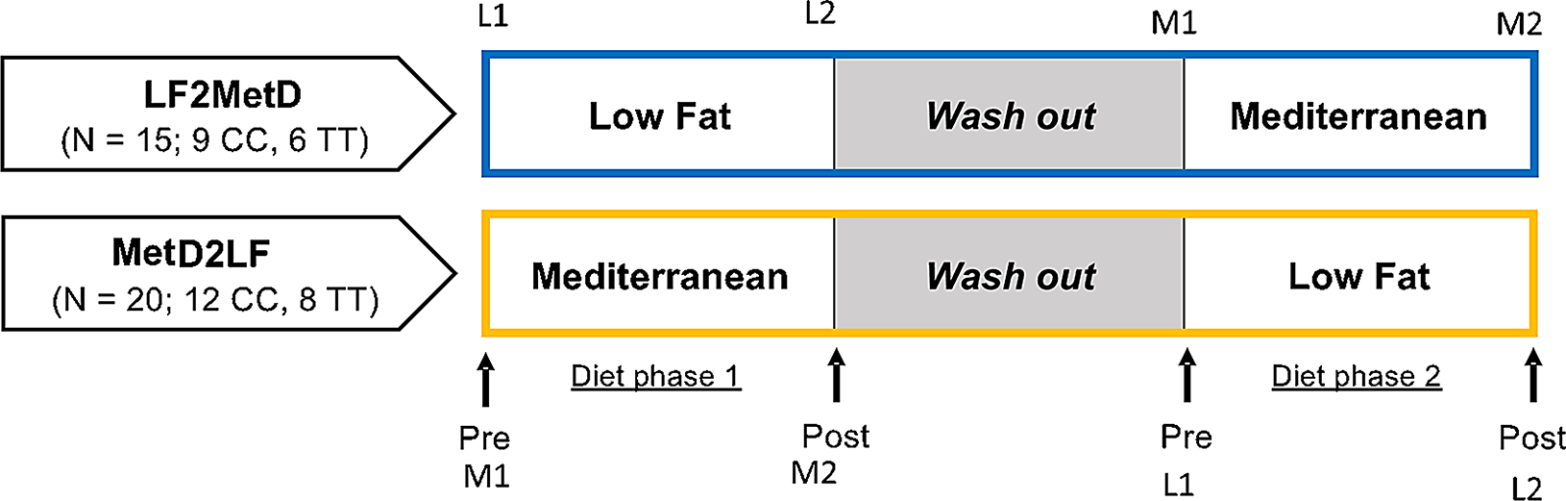
Each diet phase spanned 1 week, separated by a 1-week washout. Eligible participants were randomly assigned to one of two intervention groups: LF2MetD which followed the low-fat (LF) diet first and the Mediterranean (MetD) diet second, or MetD2LF which followed the MetD diet first and the LF diet second. Participants completed “pre” and “post” assessments for both diet phases. The main outcome was the change (post-pre) in outcomes following each diet phase

This genetics-based dietary intervention study was conducted at the JM-USDA Human Nutrition Research Center on Aging at Tufts University from 15 Feb 2018 to 7 Feb 2020. A detailed description of the study design, experimental procedures, and study participants has been described [30]. Briefly, we used a randomized crossover design with subjects selected according to *TCF7L2* genotype (*n* = 35; 21 CC/14 TT; 20 males/15 females). Each participant consumed one of two diets for 1 week: the LF diet or the MetD diet, followed by at least a 10-day washout period and a 1-week replacement diet (see Fig. 1). Thus, the intervention trial was designated into two intervention groups: LF first, then washout and ending with MetD diet (LF2MetD), and MetD first, then washout followed by LF diet (MetD2LF). The objective was to evaluate the impact of two diets on changes in plasma TRL concentrations in subjects selected according to *TCF7L2* genotype. The inclusion criteria were male and female (not pregnant) > 18 years, body mass index ranging 27–34 kg/m^2^ to represent the 50th to 75th percentile of the USA population, CC or TT genotype at rs7903146-*TCF7L2* and willingness to follow dietary habits. Exclusion criteria were liver disease, renal insufficiency, excessive alcohol consumption, chemical vapor deposition, angina, uncontrolled T2D or other significant endocrine disease, uncontrolled hypertension, pancreatitis, lipid lowering or diabetes medications, smoking; inability to follow any experimental diet or perform sampling required for this study. The sample size was calculated to detect a minimum effect of 2.5-fold change with 80% power, a specific FDR of 20% is *n* = 20 per intervention group (40 for the entire study, see Supplement 1).

#### Testing on pre- and post-intervention visits

Height was measured in centimeters with the sock foot on a solid floor. Weight was measured in kilograms on a digital scale with shoes off. Waist and hip circumference were measured in centimeters using a standardized protocol [30]. Other data collected include pulse and oral temperature. Blood samples were collected at pre- and post-intervention for metabolomics analysis (see Fig. 1).

#### Experimental diet

The intervention diets were a Mediterranean diet (MetD) or a low-fat diet (LF), each totaling 2200 kcal, designed to maintain body weight. The MetD diet contained the following percentages of calories: 41% fat (9% of total energy from saturated fat), 42% carbohydrates, 17% protein, and 26 g of total dietary fiber. The LF diet contained the following percentages of calories: 30% fat (9% of total energy from saturated fat), 53% carbohydrates, 17% protein, and 23 g of total dietary fiber.

An example MetD included breakfast as low-fat granola cereal and fresh banana, lunch as Greek wrap (tortilla, hummus, vegetables), snack as red seedless grapes, and dinner as lentils with olives and feta cheese. An example LF diet consisted of breakfast as total whole grain cereal with milk and fresh banana, lunch as chef salad with balsamic vinegar and mandarin orange slices, dinner as turkey breast, sweet potato and creamy applesauce with cinnamon.

#### Intervention

All meals were fully cooked and pre-packaged, and then dispensed as frozen, refrigerated, or shelf-stable foods for at-home consumption. Participants were advised to visit the HNRCA three times per week in order to pick up their packaged meals. Participants were expected to eat one meal on site when they arrived, and pick up the remainder of the meals (breakfast, lunch, and dinner) to take home. They received a food checklist with each day’s meals as a guide to organize the various meals. Duration of each intervention was 7 days. Participants were randomly assigned one of the diets, and after a 1–2 week washout period were placed into the other diet group for the second 7-day intervention. The 7-day intervention period was chosen as a short-term dietary challenge to observe initial metabolic changes. The typical washout period was 10 days, during which time participants consumed their own food and their usual diet. Compliance was monitored by providing all meals to participants and checking for adherence through self-reports.

#### *TCF7L2* genotyping

Saliva samples were collected from participants at least one hour after meal using a DNA Genotek (catalog number OGR-500) (DNA Genotek, Ottawa, Ontario, Canada) and genomic DNA was isolated using PrepIT*L2P Kit DNA Genotek (Cat. No. UF03) according to the supplier’s protocol. The *TCF7L2* T/C rs7903146 SNP was genotyped by TaqMan assay (Applied Biosystem QuanStudio 6 Flex) as described [31].

### Metabolomics analysis

Participants were asked to fast overnight for at least 12 h before and after each dietary intervention period prior to drawing venous blood (50 ml). Samples were stored on ice immediately after collection and during transport to the on-site laboratory. Blood was centrifuged at 3000 × *g* for 15 min at 4 °C and completely processed within one hour of collection. Plasma and serum aliquots were frozen at -80 °C until analysis. An aliquot of 500 ul plasma were stored and used for metabolomic analysis.

Metabolomic analysis of plasma was performed as described [32] by Nightingale Health (Helsinki, Finland). Briefly, 85 ul of 1.5 mg/ml EDTA plasma was delivered to a high-throughput NMR metabolomics platform deployed on all participant pre- and post-intervention plasma samples. The platform quantifies 249 metabolic biomarkers, 168 of which are directly measured and 81 of which are ratios of these markers, including amino acids, fatty acids, lipids, ketone bodies and other low molecular weight metabolic biomarkers, as well as lipoproteins subclass distribution, particle size and composition. The concentrations of variously sized lipoproteins were determined by analyzing the distinct amplitudes of lipid methyl NMR signals. To calculate the average size of lipoprotein particles, the diameters of each subclass were summed and then multiplied by their respective mass percentages, which were derived from the amplitude of their methyl NMR signals [33]. All analyses were performed as batches. Batch effects and quality controls were performed on each sample, and data were returned as absolute relative concentrations (e.g., mmol/L). Each metabolite was assigned an internal identifier to facilitate tracking and analysis.

### Statistical analysis

Descriptive statistics (mean±>SD and n (%)) were used to summarize participant characteristics by intervention group (LF2MD and MD2LF) and *TCF7L2* genotype. Characteristics were compared between intervention groups or genotypes using t-test or Fisher’s exact tests and across intervention groups and genotypes, using two-way ANOVA.

For analyses of TRL triglyceride concentrations, delta values (changes) for each TRL on each diet were calculated as post-intervention value (L2 for LF diet or M2 for MetD diet) minus the pre-intervention value (L1 or M1, respectively) (Fig. 1). These delta values represent the change in TRL triglyceride in response to the one-week intervention for that participant. TRL concentration mmol/L was converted to umol/L for statistical analysis.

To determine the main effect of *TCF7L2* genotype on the changes in TRL concentration, first, delta values for each diet (LF or MetD) was calculated in intervention group (LF2MetD and MetD2LF). Then, the TRL triglyceride delta values were estimated using linear mixed effect regression models with subject ID as random effect while adjusting for sex, age, BMI, and pre-intervention value. Proc GENMOD in SAS 9.4 (Cary, NC, USA) was used for this analysis. Similar models were employed for the main effect of diet on the changes in TRL concentration. To examine differential responses of TRL triglyceride by genotypes for each diet, participants were stratified by diet and then changes in TRLs between genotypes were estimated using a mixed regression model while controlling for sex, age, BMI, and pre-intervention values and subject ID. Similar analyses were done to determine the differential responses between diets within each genotype. To detect an interaction between *TCF7L2* genotype and diet on changes in the TRL profile, an interaction term between *TCF7L2* genotype and diet was included in the mixed linear regression models while controlling for other potential confounding factors (sex, age, BMI, repeated measures of each participant). Statistical analysis was conducted using either R (version 4.2.2) [34], RStudio IDE (version 2023.03.1 + 446) or SAS 9.4 (Cary, NC, USA).

## Results

### General characteristics of participants at baseline

**Fig. 2:**
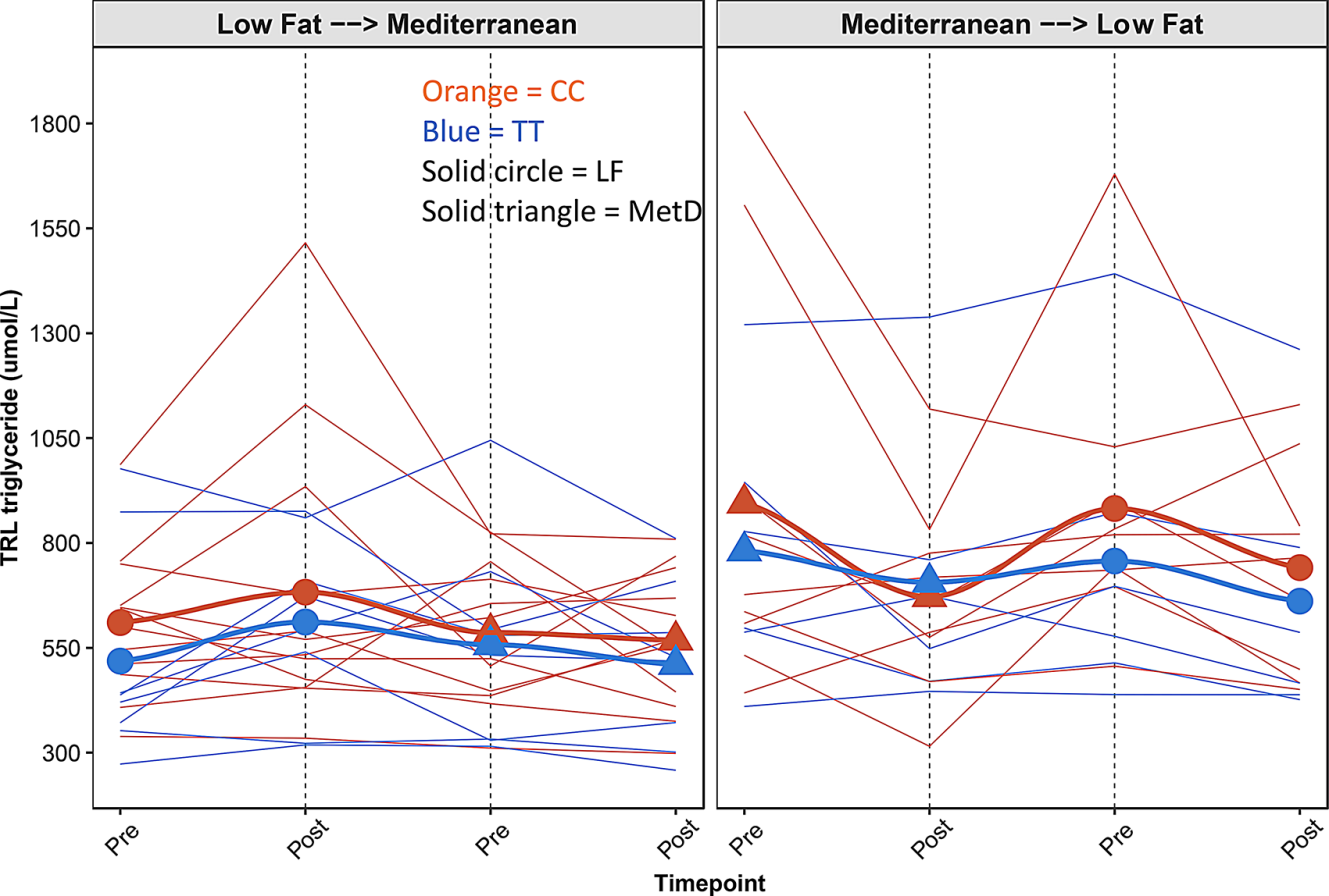
Total TRL triglyceride across study time points by intervention group and *TCF7L2* genotype. Thin solid lines represent the total TRL-TG for each participant across the study time points. Colors represent *TCF7L2* genotype (orange = CC; blue = TT); solid shapes represent mean TRL-TG at each time point for each diet phase (circle = LF; triangle = MetD). The thick solid lines represent the average TRL-TG among all participants overtime determined from a generalized linear model

Participant characteristics at baseline are provided according to intervention group (LF2MetD vs. MetD2LF) and *TCF7L2* genotypes (TT vs. CC) in Table 1. There were no significant differences in age, BMI, or sex ratio between genotypes within intervention groups, nor between intervention groups regardless of genotypes at baseline. Additionally, there were no significant differences in cholesterol particle between diet groups or between genotypes. Similarly, there were no significant differences in total TRL particles (Fig. 2), or VLDL sub-class (extra large (XL), large (L), medium (M), small (S), very small (XS)) concentrations between genotypes nor intervention groups (Table 1). As this trial was a short-term, one-week intervention for each diet, we focused on triglyceride-rich (TG-rich) lipoprotein changes in response to randomized intervention with two diets.

**Table 1:** Participants characteristics at baseline by intervention group and *TCF7L2* genotype

	LF2MetD			MetD2LF			Global *P*^3^
	CC genotype	TT genotype	*P* ^2^	CC genotype	TT genotype	*P* ^2^	
*n*	12	8		9	6		
Sex, male	8 (67%)	6 (75%)	> 0.99	4 (44%)	2 (33%)	> 0.99	0.328
Age, years	49.9 ± 17.9	46.5 ± 19.9	0.702	47.2 ± 16.9	37.7 ± 22	0.391	0.636
BMI, kg/m^2^	30.6 ± 2	29 ± 2.3	0.139	30.6 ± 1.9	31.1 ± 2	0.653	0.228
Cholesterol, mmol/L^4^							
Total cholesterol	4.69 ± 0.83	4.2 ± 0.73	0.179	4.9 ± 1.02	5.24 ± 0.96	0.524	0.175
VLDL cholesterol	0.63 ± 0.18	0.52 ± 0.16	0.189	0.75 ± 0.31	0.8 ± 0.3	0.735	0.109
LDL cholesterol	2.9 ± 0.74	2.42 ± 0.53	0.105	3.02 ± 0.74	3.34 ± 0.78	0.439	0.119
HDL cholesterol	1.37 ± 0.21	1.36 ± 0.31	0.905	1.35 ± 0.22	1.35 ± 0.17	0.985	0.996
Triglyceride, mmol/L	0.85 ± 0.24	0.73 ± 0.33	0.387	1.27 ± 0.78	1.09 ± 0.44	0.579	0.113
TRL triglyceride, mmol/L							
Total TRL	0.61 ± 0.17	0.52 ± 0.26	0.397	0.89 ± 0.49	0.78 ± 0.33	0.598	0.091
XL VLDL	0.07 ± 0.04	0.05 ± 0.05	0.397	0.12 ± 0.1	0.09 ± 0.06	0.498	0.108
L VLDL	0.12 ± 0.05	0.1 ± 0.07	0.488	0.19 ± 0.13	0.16 ± 0.08	0.589	0.104
M VLDL	0.2 ± 0.06	0.17 ± 0.09	0.433	0.29 ± 0.15	0.27 ± 0.11	0.683	0.084
S VLDL	0.1 ± 0.02	0.09 ± 0.03	0.422	0.15 ± 0.07	0.13 ± 0.05	0.537	0.077
XS VLDL	0.05 ± 0.01	0.04 ± 0.01	0.249	0.06 ± 0.02	0.05 ± 0.02	0.655	0.235
IDL	0.07 ± 0.01	0.07 ± 0.01	0.212	0.08 ± 0.03	0.08 ± 0.02	0.866	0.426

^1^Values are mean ± SD or *n* (%), unless otherwise specified. BMI, body mass index; IDL, Intermediate density lipoprotein; LF2MetD, Low fat to Mediterranean diet order; MetD2LF, Mediterranean to Low fat diet order; TRL, triglyceride-rich lipoprotein; VLDL, very low density lipoprotein

^2^P for difference between genotype for each diet group from T-test or Fischer’s exact test for continuous or categorical variables, respectively

^3^P for global difference across 4 groups (by diet group and genotype) from two-way ANOVA

^4^Data from ”Clinical LDL Cholesterol’ variable in Nightingale platform; provides concentrations consistent with routine clinical chemistry and the Friedewald equation

### Main effects of *TCF7L2* genotype and diet on TRL triglyceride

**Fig. 3:**
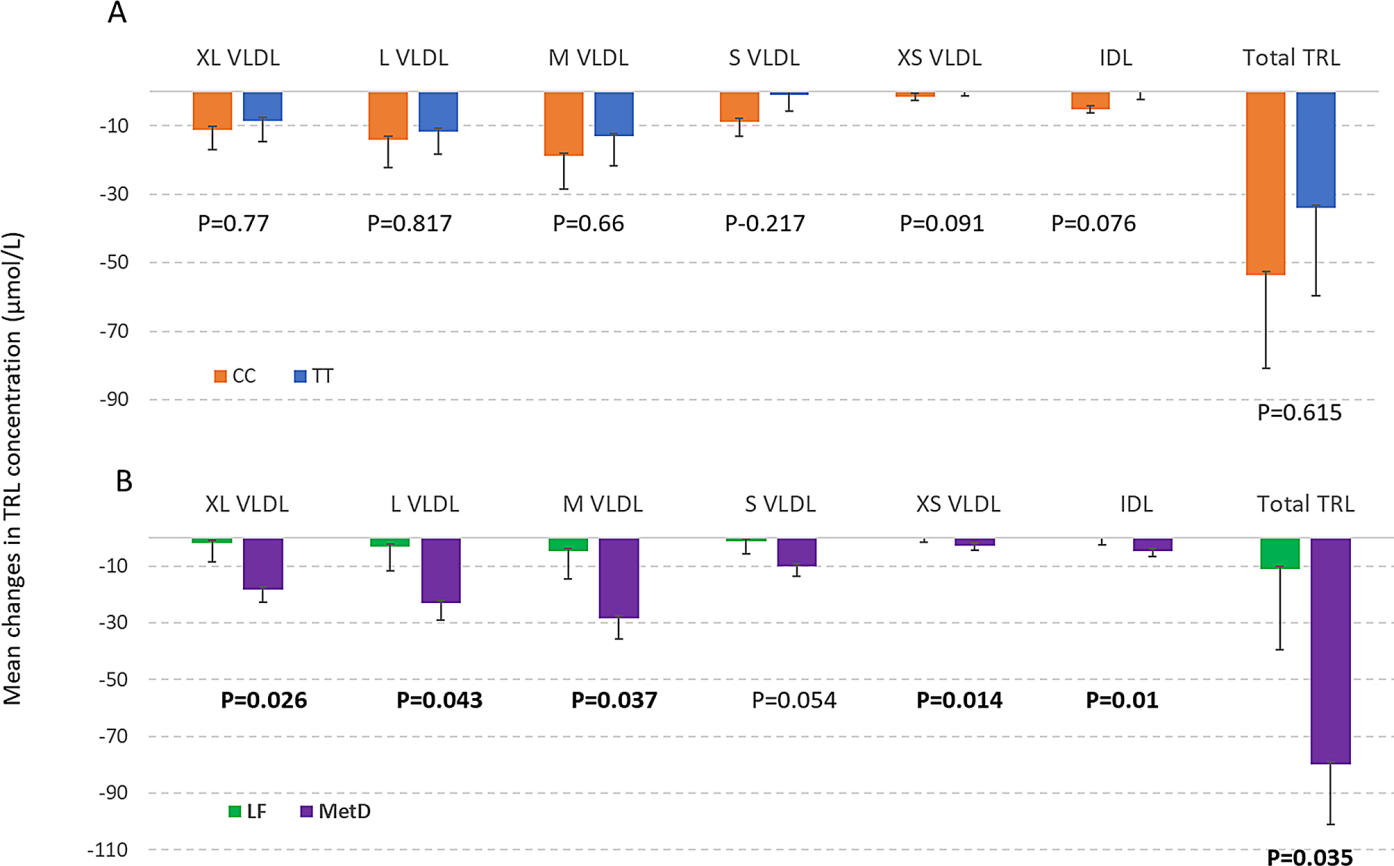
Mean (± SE) changes in TRL triglyceride concentration (µmol/L) after 1 week on a LF or MetD diet, by *TCF7L2* rs7903146 (CC vs. TT) genotype (**A**, orange bar = CC, blue bar = TT), and by diet (**B**, green bar = LF, purple bar = MetD). P-values are for the main effect of each variable from linear mixed effects regression. Least square means were estimated using linear mixed effect regression models, adjusted for pre-measured value, age, sex, BMI and a random effect for participant ID to account for correlated values within individuals

First, we examined the main effect of *TCF7L2* genotype on the TRL changes by estimating the delta value for each TRL particle by genotype on TRLs and controlling for the measurement at pre-intervention for possible spillover effect from the previous diet or habitual diet and other potential cofounding factors (age, sex, BMI). As shown in Fig. 3A, there were no significant differences in six TRLs nor in total TRL between *TCF7L2* (CC vs. TT) genotypes.

Next, we evaluated if the main effects of diets on TRL triglyceride concentrations using similar models as for *TCF7L2* genotype. As shown in Fig. 3B, there were significant differences in magnitude of TRL responses between the two diets. The MetD diet had stronger effects on reducing TRLs (extra-large VLDL, large VLDL, medium VLDL, very small VLDL, and IDL), especially total TRL, compared to the LF diet (Fig. 3B).

### Differential responses in TRL triglyceride between *TCF7L2* genotype for each diet

**Fig. 4:**
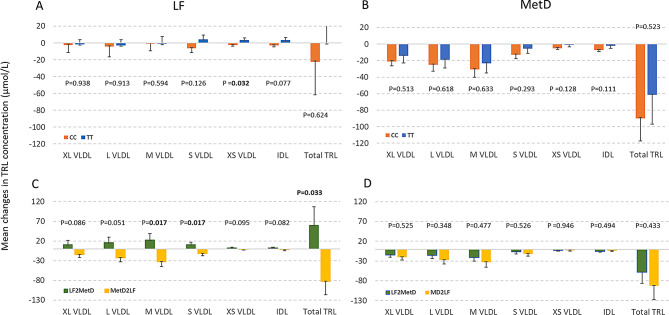
Mean (± SE) changes in TRLs by *TCF7L2* genotype and by intervention group based on adjusted linear mixed effects regression models, stratified by diet. (**A**): TT vs. CC within LF diet, (**B**): TT vs. CC within MetD, orange bar = CC and blue bar = TT. (**C**): MetD2LF vs. LF2MetD within LF, (**D**): MetD2LF vs. LF2MetD within MetD diet, dark green bar = LF2MetD, yellow bar = MetD2LF

Because diet can exert different effects on TRL concentration, we next sought to determine if the effects of *TCF7L2* genotype differed for each diet. Under the LF diet, TT carriers showed an increase in XS VLDL (*P* = 0.032) and a nominal increase in IDL (*P* = 0.077) when compared to CC carriers (Fig. 4A; Table 2). In addition, in the intervention group that started with the LF diet then proceeded to the MetD diet after washout (LF2MetD), there was a tendency toward increased TRLs compared to the intervention group (MetD2LF) that began with the MetD diet before transitioning to the LF diet (Fig. 4C). On the other hand, under the MetD, participants with both genotypes showed similar decreases in all TRL triglycerides, though there were no significant differences between genotypes (Fig. 4B). Furthermore, there was no significant difference in TRLs between the two intervention groups (Fig. 4D, LF2MetD and MetD2LF).

**Table 2:** Effects of *TCF7L2* genotype on change in TRL particle TG from adjusted linear mixed effects regression models, stratified by diet^1^

	LF				MetD			
	Genotype effect (TT vs. CC)		Order effect		Genotype effect (TT vs. CC)		Order effect	
TRL triglyceride (umol/L)	Beta (SE)	P	Beta (SE)	P	Beta (SE)	P	Beta (SE)	P
XL VLDL	0.77 (9.81)	0.938	25.57 (14.87)	0.086	6.65 (10.17)	0.513	5.59 (8.79)	0.525
L VLDL	1.45 (13.29)	0.913	38.46 (19.72)	0.051	6.31 (12.67)	0.618	11.58 (12.34)	0.348
M VLDL	8.34 (15.66)	0.594	55.40 (23.15)	0.017	7.11 (14.86)	0.633	11.63 (16.34)	0.477
S VLDL	10.90 (7.13)	0.126	22.64 (9.52)	0.017	7.32 (6.96)	0.293	4.84 (7.62)	0.526
XS VLDL	5.98 (2.79)	0.032	4.18 (2.50)	0.095	4.26 (2.80)	0.128	-0.165 (2.43)	0.946
IDL	6.44 (3.65)	0.077	4.84 (2.78)	0.082	5.36 (3.36)	0.111	-1.93 (2.82)	0.494
Total TRL	22.10 (45.06)	0.624	142.43 (66.92)	0.033	28.33 (44.32)	0.523	34.50 (44.02)	0.433

Values are beta (SE) from multivariable linear regression models, adjusted for pre-measured value, age, sex, and BMI, stratified by diet (LF or MetD). Bolded P-values were statistically significant at *P* < 0.05

^1^P-values are for the main effect of each variable

### Differential responses in TRL triglyceride between diets for each *TCF7L2* genotype

**Fig. 5:**
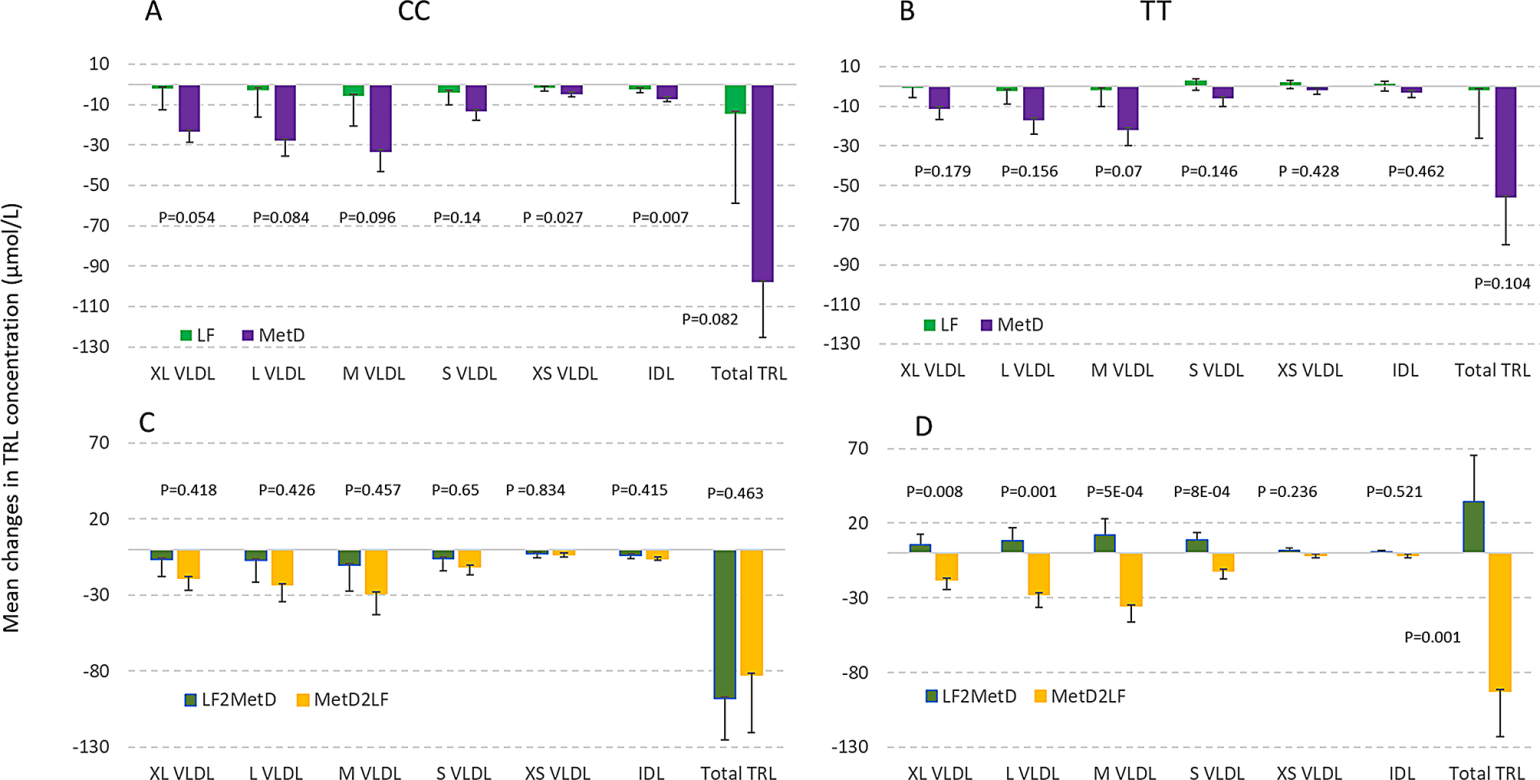
Mean change (± SE) in TRL particle TG by diet based on adjusted linear mixed effects regression models, stratified by genotype. (**A**): LF vs. MetD within CC, (**B**): LF vs. MetD within TT, green bar = LF, purple bar = MetD; (**C**): MetD2LF vs. LF2MetD within CC, (**D**): MetD2LF vs. LF2MetD within TT, dark green bar = LF2MetD, yellow bar = MetD2LF

For each genotype, the LF and MetD diets could have different effects on TRLs, and this was examined with the following approach. As shown in Fig. 5A; Table 3, for CC carriers, the MetD significantly showed a trend toward reducing very small VLDL (*P* = 0.027) and IDL (*P* = 0.007) when compared to the LF diet. Furthermore, there were no significant differences in TRLs between the two intervention groups (LF2MetD vs. MetD2LF). For TT carriers, there was no significant difference in TRL reduction between the two diets. Strikingly, the intervention group MetD2LF displayed greater reductions in extra large VLDL (*P* = 0.008), large VLDL (*P* = 0.001), medium VLDL (*P* = 5E-04), small VLDL (*P* = 8E0-4) and total TRL (*P* = 0.001), whereas LF2MetD tended to increase TRL levels (Fig. 5D). These findings demonstrate the significant cardiovascular benefits of the Mediterranean diet for TT genotype carriers.

**Table 3:** Effects (beta ± SE) of two diets on change in TRL particle TG from adjusted linear mixed effects regression models, stratified by *TCF7L2* genotype^1^

	CC Genotype				TT Genotype				*P* interaction^2^
	Diet effect (LF vs. MetD)		Order effect		Diet effect (LF vs. MetD)		Order effect		
TRL triglyceride (umol/L)	Beta (SE)	P	Beta (SE)	P	Beta (SE)	P	Beta (SE)	P	
XL VLDL	21.21 (10.97)	0.054	12.25 (15.11)	0.418	10.58 (7.88)	0.179	23.39 (8.83)	0.008	0.341
L VLDL	25.15 (14.56)	0.084	16.35 (20.52)	0.426	14.53 (10.25)	0.156	35.95 (10.96)	0.001	0.486
M VLDL	27.53 (16.54)	0.096	18.61 (25.02)	0.457	20.19 (11.13)	0.07	47.36 (13.68)	5.00E-04	0.671
S VLDL	9.35 (6.34)	0.14	5.03 (11.06)	0.65	9.26 (6.37)	0.146	20.63 (6.17)	8.00E-04	0.866
XS VLDL	3.01 (1.36)	0.027	0.61 (2.92)	0.834	3.91 (4.93)	0.428	3.36 (2.83)	0.236	0.806
IDL	4.65 (1.72)	0.007	2.16 (2.65)	0.415	4.42 (6.01)	0.462	1.83 (2.84)	0.521	0.912
Total TRL	82.51 (48.07)	0.082	52.43 (71.42)	0.463	54.20 (33.29)	0.104	126.56 (38.78)	0.001	0.548

Values are beta (SE) from multivariable linear regression models, adjusted for pre-treatment value, age, sex, and BMI, stratified by genotype. Bolded P-values were statistically significant at *P* ≤ 0.05

^1^P-values are for the main effect of each variable

^2^P interaction - P-values for interaction between genotype and diet on TRLs

### Interaction between diet and *TCF7L2* genotype on TG-rich particles

Given the observed differential response to LF and MetD diet on TRL according to *TCF7L2* genotypes, we then examined whether the interaction between *TCF7L2* and diet would alter TRL concentrations while controlling for other potential common factors. As shown in Table 3, the interaction between *TCF7L2* and diet on TRLs did not reach significance.

We also examined the effects of the two diets on plasma ApoB and cholesterol in the different subclass VLDL for the two genotypes (see Supplement 1). There were no significant differences in ApoB nor TRL cholesterol concentrations between two intervention groups (LF2MetD and MetD2LF), nor between genotypes within each intervention group (all *P* > 0.05 in Additional Table 1). Additional Fig. 1 shows a lack of significant main effects of genotype or diet on ApoB or TRL cholesterol changes in linear mixed effects regression models, with the exception of XL VLDL and L VLDL which decreased significantly more following MetD, compared to LF, diet on average (*P* = 0.035 and 0.048, respectively). As shown in Additional Fig. 2, there were no significant differences in effects of diet on ApoB nor TRL cholesterol concentrations between *TCF7L2* genotypes.

## Discussion

Previous studies reported that the Mediterranean diet diminished the adverse effects of the TT genotype at rs7903146 on the risk of type 2 diabetes and stroke [6, 7]. To characterize the biological mechanism underlying this benefit, we conducted a short-term randomized crossover intervention trial with low-fat diet and Mediterranean diet on 35 participants with two genotypes (CC and TT) at rs7903146 in *TCF7L2*. We observed significant differences in the triglyceride-rich lipoprotein profile between *TCF7L2* genotype in response to these two diets. The MetD diet significantly reduced VLDL levels compared to the LF diet. Under the LF diet, TT carriers exhibited an increase in small VLDL particles, compared to CC carriers. In contrast, the MetD diet neutralized that shift in VLDL. In addition, there was an order effect such that there was a stronger effect on the triglyceride-rich lipoprotein profile when the LF diet was first than when the MetD was the initial diet.

TG-enriched VLDL secreted from liver functions as transporters of TG and cholesterol in lipid metabolism [14, 15, 35]. VLDL circulate in the blood for about 4 h before progressive conversion to IDL and LDL [35]. Accumulating evidence supports the connection that elevated levels of fasting larger VLDL increase the risk of cardiometabolic diseases, including T2D and stroke [17, 19, 22]. In this study, our results illustrated that the MetD diet reduced larger VLDL– extra-large, large and medium– significantly more than the LF diet. This demonstrated an overall beneficial effect of the MetD in lowering risk of cardiometabolic diseases. While no significant genotype effect of *TCF7L2* was observed, when consuming the LF diet, TT carriers displayed significantly increased very small VLDL concentrations than CC carriers. Elevated very small VLDLs are also more likely to be atherosclerotic because they easily penetrate the endothelium of blood vessels and redeposit in the artery walls, causing inflammation and plaque buildup [20, 21]. On the other hand, this difference between TT and CC carriers in very small VLDL disappeared under MetD diet, suggesting that the MetD diet reduced very small VLDL concentrations in TT carriers. As TT carriers have impaired insulin sensitivity [36], MetD diet could enhance insulin sensitivity [37] that leads to increased lipoprotein lipase activity [38] and hence neutralize the elevated TRL concentration. Such speculation remains to be tested in a future study.

The T allele at rs7903146 is known to be a risk allele for T2D and cardiovascular disease as well as metabolic syndrome [2, 6, 7]. Most studies report that T allele carriers display elevated TG and TG-rich VLDL [24, 39, 40]. Furthermore, in a recent report, T-allele carriers showed a higher postprandial TG after a fat challenge test (62.5 g fat, 24.75 g 5.3 g protein, 240 mg cholesterol) than CC carriers [41]. In addition, T2D and pre-diabetes groups exhibited significantly higher expression of *TCF7L2* than the non-disease group in visceral adipose tissue of Asian Indians [41]. However, differential results in the postprandial response were observed in another intervention trial [23], where CC homozygotes showed a higher postprandial response to a test meal (34% fat, 47% carbohydrate, 19% protein) than TT carriers. In the present intervention, CC carriers tended to have higher TRL concentrations, although not significantly, especially when consuming the LF diet, regardless of the intervention course (Fig. 2). However, TT carriers tended to have higher TRL when consuming MetD diet first in the MetD2LF intervention group (Fig. 2). This inconsistency may be explained by evidence that *TCF7L2* interacts with dietary fat intake on TG and VLDL. We previously have reported that *TCF7L2* genotypes display interactions with PUFA-intake on TG and VLDL, as well as postprandial responses in VLDL and TG [24]. When PUFA intake was low (< 7.36% of total energy), T allele carriers showed diminished or no differences in total VLDL or postprandial TG response than CC carriers. In contrast, when the PUFA intake was greater than 7.26% of total energy, T allele carriers showed higher postprandial responses in total VLDL and TG than CC carriers. Genotype by PUFA interactions may explain the discordance of T alleles on TG, VLDL observed in the current and other studies [23].

Under the LF diet, there was a significant difference in TRL triglyceride responses based on the order in which the diet was followed. The LF2MetD group showed increased TRL levels compared to the MetD2LF group, with LF2MetD trending toward increased TRLs, an indication of increased risk of cardiometabolic diseases, whereas MetD2LF decreased TRLs. This difference, based on the order effect of the two diets, could reflect a spillover effect of the first diet to the second diet, suggesting that a one-week washout is not sufficient to prevent this spillover effect. For example, the first diet can induce epigenetic changes that affect the baseline of the second dietary intervention [42]. Furthermore, the order effect was only evident within the LF diet (Table 2) or TT carriers (Table 3), suggesting a beneficial effect of MetD in CVD prevention in TT carriers by reducing TRL concentrations.

Interactions between *TCF7L2* genotype and diet have been reported on metabolic disease, such as type 2 diabetes and obesity [43, 44]. It is puzzling that when stratified by diet, differential responses in TRL profiles between genotypes were observed, but the effect of a diet interaction with *TCF7L2* was not significant. This can be explained in any of three ways. First, stratification was performed by diet, which empowered the analyses to detect main effects of genotype to influence TRLs. In contrast, interactions between genotype and diet are often not linear, so the power to detect interactions is much lower than the power to detect main effects [45]. Second, the small sample size also limits the ability to detect genotype by diet interactions. Simulations show that, assuming the interaction is the same size as the main effect, the sample size required to detect the interaction is approximately four times the sample size required to detect its main effect [46]. Third, spillover effects observed from the prior intervention diet or habitual diets (before entering the intervention study) also may affect the detection of interactions between *TCF7L2* genotype and diet.

This study has some limitations. First, for a randomized crossover and short-term clinical trial, the 10-day washout period may have been insufficient in duration, resulting in spillover effects on subsequent diets. We acknowledged the varied length of the washout period across all participants and lipid metabolism might stabilize over a longer period, so a longer fixed washout period might provide more robust results. Our analysis focused on changes between pre- and post-intervention and controlled for pre-intervention values, minimizing spillover effects on the later diet. Future intervention trials will require longer washout periods. Second, the 7-day intervention period was chosen as a short-term dietary challenge to observe initial metabolic changes. While longer periods would provide more information on sustained effects, this study was designed to capture acute dietary responses, which offer valuable data for understanding early metabolic changes related to genotype-diet interactions. Third, the small sample size plus the spillover effect of prior diet may have limited the ability to detect a genotype by diet interaction on TRL changes in the crossover intervention. Fourth, diets consumed during the washout period may not have reflected the habitual diet prior to entering the study. Finally, the study design used a BMI range of 27 to 34, chosen for participants at higher metabolic risk. While it is possible that results may differ in individuals with a lower or healthier BMI, our study focused on a population with a BMI in the overweight to obese range, which better reflects the target population for dietary interventions focusing on type 2 diabetes and obesity.

## Conclusion

In summary, TT genotype carriers experienced greater improvements in their triglyceride-rich lipoprotein profiles when adhering to a Mediterranean diet. This effect on TRLs may lower their risk of cardiovascular disease. More broadly, this study shows an important aspect of how the Mediterranean diet can exert differential effects on metabolism based on genetic differences.

## Electronic supplementary material

Below is the link to the electronic supplementary material.

Supplementary Material 1

## Data Availability

No datasets were generated or analysed during the current study.
