# Supplementary material for: Changes in triglyceride-rich lipoprotein particle profiles in response to one-week on a low fat or Mediterranean diet by TCF7L2 rs7903146 genotype: a randomized crossover dietary intervention trial
Source: Genes Nutr. 2025 Mar 6;20:4. doi: 10.1186/s12263-025-00763-y (PMC11884055; doi:10.1186/s12263-025-00763-y)
Supplement: Supplementary file 1 — Supplementary Material 1 [file 12263_2025_763_MOESM1_ESM.docx]

Supplement to the manuscript entitled “Changes in Triglyceride-Rich Lipoprotein Particle profiles in response to One-week on a Low Fat or Mediterranean Diet by *TCF7L2* rs7903146 genotype: A Randomized Crossover Dietary Intervention Trial” by Lai *et al*.

ClinicalTrials.gov: https://[www.clinicaltrials.gov/study/NCT03458494.](http://www.clinicaltrials.gov/study/NCT03458494)

**Study Design**: For this genetics-based dietary intervention study, which was conducted at the JM-USDA Human Nutrition Research Center on Aging at Tufts University (HNRCA) from 15 Feb 2018 to 7 Feb 2020. Inclusion criteria were men and women aged 18 years or older, women who were not pregnant, a BMI ranging between 27 and 34 that includes the 50th to 75th percentile of the USA population. Exclusion criteria were unexplained elevation in serum transaminases (i.e. >1.5 times the upper limit of normal) or evidence of active liver disease, severe renal dysfunction (serum creatinine > 2.0mg/dL), alcohol consumption above 2 drinks/day, preexisting cardiovascular disease, stable exertional angina pectoris requiring sublingual nitroglycerin within the prior 3 months, uncontrolled T2D (fasting glucose >126 mg/dl) or other significant endocrine disease, uncontrolled hypertension (systolic blood pressure

>180 mmHg or diastolic blood pressure >100 mmHg), pancreatitis within 1 year, taking lipid lowering or diabetes medications, smoking, pregnancy, BMI below 27 or greater than 34 kg/m^2^, any of drug abuse, extreme dietary habits, multiple food allergies, extreme levels of physical or athletic activity, or by changes in body weight >20 lbs during the last 6 months, inability to follow the experimental diets (including being vegetarian) or to perform the sampling required for this study, thyroid diseases, and use of omega-3 supplements (unless discontinued 1 month prior to the beginning of the study). Persons expressing interest in the study were screened for general eligibility, and each provided signed consent forms, anthropometric measures, a saliva sample for DNA genotyping, completed general health questionnaire and medical history, and a blood sample. The study was approved by the Institutional Review Board at Tufts University (12691), and adhered to the ethical principles of the Helsinki Declaration of 1975 as revised in 1983.

**Sample size calculation**: Based on biomarker data from a previous study, we calculated the standard deviation associated with interaction effects in an ANOVA model. Using this estimate of standard deviation, along with >80% power, a specified FDR of 20%, 75% proportion of null hypothesis effects, for a minimum detectable effect size of 2.5-fold change, we require a sample size of n=20 subjects per intervention group (40 for the entire study). Sample size calculations have incorporated FDR using the R package “ssize.fdr.” The observed effect size of 2.5 represents an interaction effect between TT and CC genotypes within diet type.

**Genotyping:** Saliva for DNA isolation and analysis was collected with the DNA Genotek (#OGR-500) (DNA Genotek, Ottawa, ON, Canada) per vendor’s protocol. Participants waited at least 1 hr after eating before expectorating. DNA was purified for PCR analysis using the PrepIT*L2P kit (DNA Genotek #UF03). In brief, whole sample mixed with lysis buffer was incubated at room temperature for 3 hrs. Then 500 µl of the mix was combined with 20 µl of L2P in a 1.5 ml tube, placed on ice for 10 mins, and then spun at 15000 x *g* for 10 mins. The supernatant was transferred to a new tube and mixed with 600 µl of 100% ETOH at room temperature. The tube was inverted gently 10 times, then spun at room temperature for 10 min.

DNA pellets were washed with 250 µl of 70% ETOH and dissolved in 100 µl of TE. The TCF7L2 T/C rs7903146 SNP was genotyped with the TaqMan assay (Applied Biosystem QuanStudio 6 Flex, ThermoFisher Scientific, Waltham, MA, USA) as reported [31].

A goal in forming the study group was 40 participants, comprised of 20 of each genotype CC and

TT. Because the group size was small and because TT is the rarer genotype at the rs7903146 variant (~8%-11% of the US population), it was important to match TT and CC individuals by certain influential characteristics. This was accomplished by matching age and sex.

**Intervention:** The intervention diets were either Mediterranean (MetD) or low-fat (LF), each of 2200 total kilocalories and designed to maintain body weight. Participants with the same genotype (TT or CC) were randomized to assign to LF first or Med diet first. The Med-based diet had the following percent calories: 41% fat (9% total energy from saturated fat), 42% carbohydrate, 17% protein, and 26 g total dietary fiber. The LF-based diet had the following percent calories: 30% fat (9% total energy from saturated fat), 53% carbohydrate, 17% protein, and 23 g total dietary fiber.

An example Med diet included breakfast as low-fat granola cereal and fresh banana, lunch as Greek wrap (tortilla, hummus, vegetables), snack as red seedless grapes, and dinner as lentils with olives and feta cheese. An example LF diet consisted of breakfast as total whole grain cereal with milk and fresh banana, lunch as chef salad with balsamic vinegar and mandarin orange slices, dinner as turkey breast, sweet potato and creamy applesauce with cinnamon.

All meals were fully cooked and pre-packaged, and then dispensed as frozen, refrigerated, or shelf-stable foods for at-home consumption. Participants were advised to visit the HNRCA three times per week in order to pick up their packaged meals. Participants were expected to eat one meal on site when they arrived, and pick up the remainder of the meals (breakfast, lunch, and dinner) to take home. They received a food checklist with each day’s meals as a guide to organize the various meals. Duration of each intervention was 7 days. Participants were randomly assigned one of the diets, and after a 1-2 wk washout period were placed into the other diet group for the second 7-day intervention. The typical washout period was 10 days, during which time participants consumed their own food and their usual diet. Compliance was monitored by providing all meals to participants and checking for adherence through self-reports.

**Testing performed during visits at the beginning and end of the intervention:** Physical examinations included height and weight. Height was measured in cm in stocking feet on a firm floor. Weight was measured in kg without shoes on a digital scale. Waist and hip circumferences were measured in cm using standardized protocols. Other data collected were pulse and oral temperature. Participants were requested to fast overnight for at least 12 hr prior to the venous blood draw (50 ml) for metabolomics analysis, taken both prior to and upon completion of each intervention. Samples were stored on ice immediately after collection and during transport to the on-site laboratory. Blood was centrifuged at 3000 x *g* for 15 min at 4°C, and fully processed within one hour of collection. Plasma and serum aliquots were frozen at -80°C until analysis. All analyses were performed as a batch. Aliquots of 500 ul were taken and stored for metabolomics analysis and other clinical measures.

**Metabolomics:** Metabolomics profiling of plasma was performed as described [32] by Nightingale Health (Helsinki, Finland). Briefly, 85 ul of plasma in EDTA at 1.5 mg/ml was passed to a high-throughput NMR metabolomics platform that was deployed on plasma samples from the beginning of and end of the intervention from all participants. That platform quantified 249 metabolic biomarkers of which 168 were directly measured and 81 were ratios of these, including amino acids, fatty acids, lipids, ketone bodies and other low-molecular-weight metabolic biomarkers, plus lipoprotein subclass distribution, particle size and composition. Each sample was evaluated for batch effects and quality control, and data were returned in absolute relative units (mmol/L, %). We assigned each metabolite an in-house identifier to facilitate tracking and analysis.

**The results of the LF and MetD diets on TRL cholesterol in the different VLDL species and ApoB for the two genotype groups**

Mixed linear regression models similar to those for TRL triglycerides were applied to examine the concentration of ApoB and TRL cholesterol. As the Nightingale metabolomics platform does not provide an additional breakdown of ApoB by VLDL subclasses, only total ApoB concentration was used for this analysis.

There were no significant differences in ApoB nor TRL cholesterol concentrations between the two intervention groups (LF2MetD and MetD2LF), nor between genotypes within each intervention group (all *P* >0.05 in Additional Table 1). Additional Figure 1 shows a lack of significant main effects of genotype or diet on ApoB or TRL cholesterol changes in linear mixed effects regression models, with the exception of XL VLDL and L VLDL which decreased significantly more following the Mediterranean, compared to Low Fat, diet on average (*P* = 0.035 and 0.048, respectively). As shown in Additional Figure 2, there were no significant differences in effects of diet on ApoB nor TRL cholesterol concentrations between TCF7L2 genotype groups.

| **Additional Table 1.** Apolipoprotein B and TRL cholesterol concentrations by intervention group and TCF7L2 genotype | | | | | | | |
| --- | --- | --- | --- | --- | --- | --- | --- |
|  | **LF2MetD** | | | **MetD2LF** | | | **Global P** |
|  | CC | TT | P2 | CC | TT | P2 |  |
| *N* | 12 | 8 | 20 | 9 | 6 | 15 | - |
| Apolipoprotein B, g/L | 841.4 ± 206.1 | 713.1 ± 155.1 | 0.131 | 905.1 ± 253.8 | 990.2 ± 262.5 | 0.546 | 0.128 |
| TRL cholesterol, mmol/L |  |  |  |  |  |  |  |
| XL VLDL | 0.02 ± 0.01 | 0.02 ± 0.01 | 0.290 | 0.03 ± 0.02 | 0.04 ± 0.02 | 0.891 | 0.081 |
| L VLDL | 0.04 ± 0.02 | 0.03 ± 0.02 | 0.364 | 0.06 ± 0.03 | 0.06 ± 0.03 | 0.93 | 0.069 |
| M VLDL | 0.1 ± 0.04 | 0.08 ± 0.02 | 0.145 | 0.1 ± 0.04 | 0.12 ± 0.04 | 0.251 | 0.131 |
| S VLDL | 0.09 ± 0.02 | 0.07 ± 0.02 | 0.099 | 0.1 ± 0.03 | 0.11 ± 0.04 | 0.701 | 0.100 |
| XS VLDL | 0.13 ± 0.03 | 0.11 ± 0.03 | 0.296 | 0.13 ± 0.04 | 0.15 ± 0.04 | 0.303 | 0.243 |
| IDL | 0.58 ± 0.13 | 0.52 ± 0.11 | 0.253 | 0.58 ± 0.13 | 0.67 ± 0.15 | 0.271 | 0.195 |
| Total TRL | 0.96 ± 0.23 | 0.83 ± 0.18 | 186 | 1.0 ± 0.28 | 1.15 ± 0.3 | 0.370 | 0.138 |

**Additional Figure 1.** Effect of diet or genotype on mean changes in ApoB and TRL cholesterol concentrations.

**
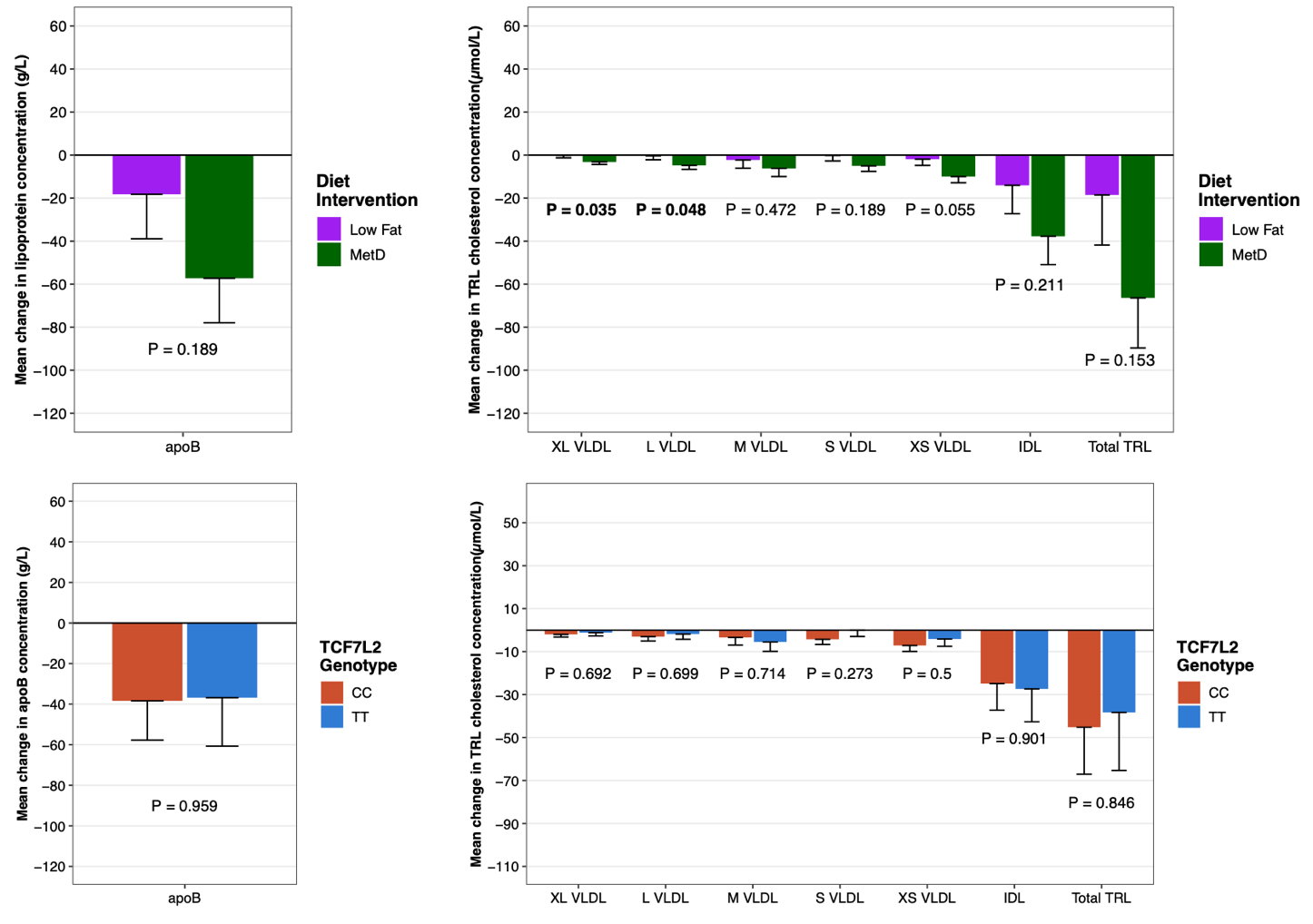
**

**+**

**Additional Figure 2.** Effect of diet and genotype on mean changes in ApoB concentrations.


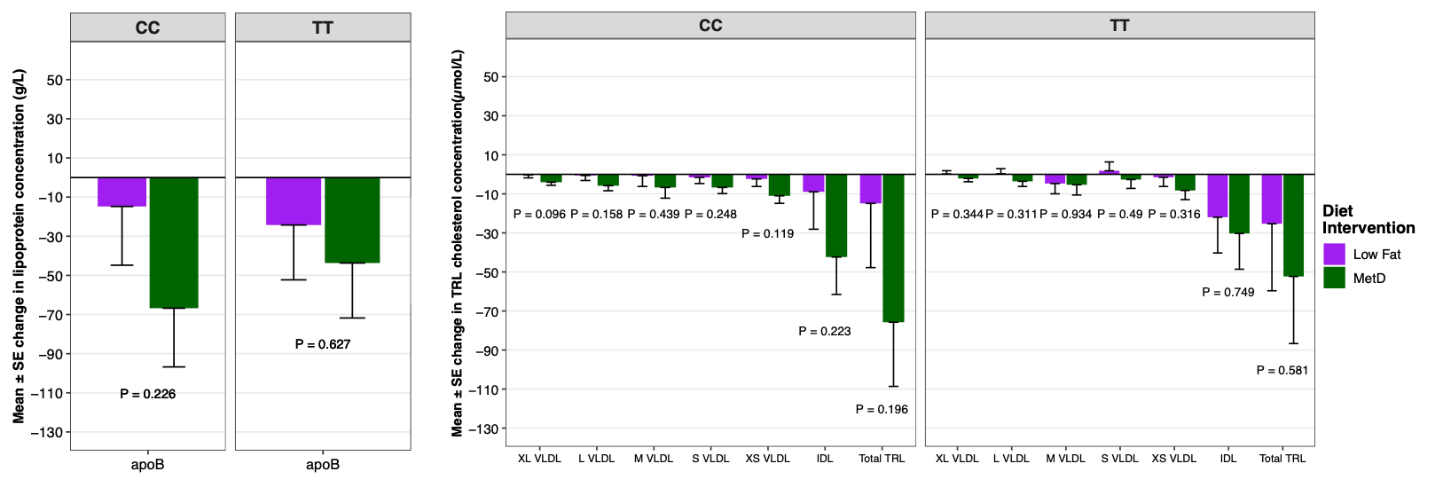


# INFORMED CONSENT TO PARTICIPATE IN RESEARCH

Jean Mayer USDA Human Nutrition Research Center on Aging (HNRCA) at Tufts University Nutrition and Genomics Laboratory

**Title of Protocol**: The TCF7L2 gene: Nutrigenetics and dietary prevention of type 2 diabetes

Principal Investigator: Jose M Ordovas, PHD Study Physician: Lisa Ceglia MD

Co-Investigators: Caren Smith, DVM

Chao-Qiang Lai, PhD Laurence Parnell, PhD Kathryn Barger, PhD Gregory Matuszek, PhD Lucia Pham, BSc

Study team telephone number: (617) 556-3135

Lisa Ceglia MD (617) 556- 3085, days

For emergencies at any time, including days, evenings, weekends and holidays, please call (617) 230-7545 to reach Dr. Ceglia, the study physician.

# Introduction

You are being invited to participate in a research study “The TCF7L2 gene: Nutrigenetics and dietary prevention of type 2 diabetes " at the Jean Mayer USDA Human Nutrition Research Center on Aging at Tufts University (HNRCA) because you completed the screening process, you were found to be eligible for the main study based on your particular form of the TCF7L2 gene as well as other criteria, and you are interested in participating.

Taking part in this research study is entirely your choice. You can decide to refuse to participate in this study. If you decide to participate in this study, you can then choose to stop taking part in the study at any time for any reason. If you refuse to participate in the study or stop being in this study, it will not affect your care or treatment outside this study, payment for your health care, or your health care benefits.

Please read all of the following information carefully. Ask Dr. Jose Ordovas, or his representative, to explain any words, terms, or sections that are unclear to you. Ask any questions that you have about this study. Do not sign this consent form unless you understand the information in it and have had your questions answered to your satisfaction.

If you decide to take part in this research study, you will be asked to sign this form. You will be given a copy of the signed form. You should keep your copy for your records. It has information, including important names and telephone numbers, to which you may wish to refer in the future.

New things might be learned during this study that you should know about. The investigators will tell you about new information that may affect your willingness to stay in this study.

You are free to withdraw from the study at any time for any reason. If you are eligible to participate and decide to be in the study, the Principal Investigator may still choose to stop your participation in this study if he thinks it is in your best medical interest. You may also be withdrawn from the study if you do not comply with study protocol or study diets, or if you do not adhere to any and all HNRCA volunteer rules and regulations. If you withdraw or are withdrawn from the study, any data collected from you before your withdrawal will still be used for the study.

As a participant in this study, your identity, medical records, and data relating to this study will be kept confidential, except as required by law. The U.S. Food and Drug Administration, which regulates investigational drug and device studies, and the study sponsor National Institute of Food and Agriculture may also look at records that identify you if applicable to the study.

If you have question about your rights as a research study subject, call the Tufts Medical Center and Tufts University Health Sciences Institutional Review Board (IRB) at (617) 636-7512. The IRB is a group of doctors, nurses, and non-medical people who review human research studies for safety and protection of people who take part in the studies. Federal law requires the IRB to review and approve any research study involving humans. This must be done before the study can begin. The study is also reviewed on a regular basis while it is in progress.

This research study has been reviewed and approved by the IRB of Tufts Medical Center and Tufts University Health Sciences.

# PURPOSE OF THE RESEARCH STUDY

Nutrients and chemicals in food are able to regulate expression of genetic elements. Gene-nutrient interaction in response to diets can increase an individual’s risk, shifting the individual from health toward the development of chronic disease. The TCF7L2 gene may either put individuals at risk for or protect from type 2 diabetes in the presence of food patterns (i.e., Mediterranean or low fat diets). The main purpose of this four-week study is to examine diet induced gene-nutrient interaction, with a focus on plasma levels of type 2 diabetes biomarkers in individuals who have either the CC or the TT form within a specific variant of the TCF7L2 gene. The (2) one-week study diets, one Mediterranean based and the other low-fat based, are separated by a (1-2) week return to your regular habitual food products.

If you choose to participate in this research study, you will be asked to visit the Metabolic Research Unit (MRU) at the HNRCA at least three days a week during each of the two weeks when you will be receiving the Mediterranean-based or the low fat-based diet.

About 300 subjects will be enrolled for this study.

# PROCEDURES

Blood collections will be taken throughout each study phase. This will allow us to evaluate how the two diets impact differently, depending on the TCF7L2 gene, on blood markers (like glucose, lipids, metabolites) known to be associated with type 2 diabetes.

Upon enrollment, you will be asked to complete two study phases (1 week each) during which time you will be given all the food items that you need to eat for each day. For this, you will be asked to come to the HNRCA at least three times per week during each of the study phases (for a total of 6 visits). During these visits your body weight, blood pressure and pulse rate will be taken; and you will be asked to eat at least one meal per week at the HNRCA and take with you all the food you will need to eat at each meal for each day until your next visit to the HNRCA. During one study phase (one-week) you will be provided with Mediterranean-based diet (the Mediterranean Diet). During the other study phase you will be provided with a Low Fat-based diet (the Low Fat diet). The order of the two study phases will be determined randomly (like flipping a coin).

The amount of food you get will be adjusted so that your body weight will stay constant. This is not a weight loss program. You will get the foods at the HNRCA. There will be a break between the two study phases. Depending on scheduling and availability, this break may be anywhere between one- and two-weeks long. During this break we will not give you any foods or beverages and you will be allowed to eat and drink whatever you like.

| **Phase 1** | **Testing on Visits: Visit 3 (Day -1)/ Visit 4 (Day 3)/ Visit 5 (Day 6)** |
| --- | --- |
| Mediterranean | Visit 3, Day -1 |
| diet | This visit will take approximately 2 hours |
| Or | 12 hour Fast (water OK) |
| Low Fat diet | Review & sign main study consent |
| Days: 1-6 | Food frequency and TFEQ questionnaires |
|  | Measure height & weight |
|  | Vital signs: oral temperature; pulse; Blood pressure #1 |
|  | Obtain second Blood pressure 2 minutes later |
|  | Measure waist & hip circumferences |
|  | Fasting blood draw 50ml (about 3.3 tablespoons) |
|  | Visit 4, Day 3 |
|  | This visit will take approximately one hour. |
|  | 12 hour Fast (water OK) |
|  | Measure weight |

|  | Vital signs: oral temperature; pulse; Blood pressure #1 Obtain second Blood pressure 2 minutes later Measure waist & hip circumferences  Fasting blood draw 50ml (about 3.3 tablespoons) |
| --- | --- |
|  | Visit 5, Day 6  This visit will take approximately one hour. 12 hour Fast (water OK)  Measure weight  Vital signs: oral temperature; pulse; Blood pressure #1 Obtain second Blood pressure 2 minutes later Measure waist & hip circumferences  Fasting blood draw 50ml (about 3.3 tablespoons) |
| **1-2 Week washout between study diets**: Own food. Usual diet. | |
| **Phase 2** | **Testing on Visits: Visit 6 (Day 13)/ Visit 7 (Day 17)/ Visit 8 (Day 20)** |
| Opposite Study Diet Days:14-20 | Visit 6, Day 13  This visit will take approximately one hour. 12 hour Fast (water OK)  Update health status & medications Measure weight  Vital signs: oral temperature; pulse; Blood pressure #1 Obtain second Blood pressure 2 minutes later Measure waist & hip circumferences  Fasting blood draw 50ml (about 3.3 tablespoons) |
|  | Visit 7, Day 17  This visit will take approximately one hour. 12 hour Fast (water OK)  Measure weight  Vital signs: oral temperature; pulse; Blood pressure #1 Obtain second Blood pressure 2 minutes later Measure waist & hip circumferences  Fasting blood draw 50ml (about 3.3 tablespoons) |
|  | Visit 8, Day 20  This visit will take approximately one hour. 12 hour Fast (water OK)  Measure weight  Vital signs: oral temperature; pulse; Blood pressure #1 Obtain second Blood pressure 2 minutes later Measure waist & hip circumferences  Fasting blood draw 50ml (about 3.3 tablespoons) |

**Study Diets:** There will be two different diets, the Mediterranean and the Low fat diet, which you will get at the Nutrition Center and you will be asked to eat all the foods we will give you. All the foods will be provided by the Nutrition Center during each of the two 1-week phases of the study.

You will be asked to visit the MRU at least three times per week during week days, unless you would like to come daily to pick up study foods. You will be required to eat at least one meal per week at the Nutrition Center and the rest of your food will be packed for you to take home.

You will be asked to eat only the food given to you during the study phases and to eat all the food provided. You will be asked to rinse and scrape the dishes/containers where the food is placed. Any remaining food will be recorded. You will also be asked to keep a checklist of what you eat and what you do not eat every day during each 1-week study phase. During your visits at the Nutrition center you will meet with study staff to assess how the study is going for you, check whether there have been any missed meals, and discuss whether you have had any problem with the diet.

It is very important that you eat only the food we give you and that you eat all of it. We will help you stick with the study diets. However, if we find that you are not adhering exclusively to the study diets your study participation will be terminated.

**Height, Weight and Anthropometrics:** Your body weight, blood pressure and pulse rate will be measured at each Center visit. For the measurement of your body weight, you will remain in light clothes. If your body weight is different from the initial weight we obtained during the start of the study by more than 2 kg, we will need to adjust the amount of calories in your diet until your body weight returns to being no more than 2 kg up or down from your initial body weight. Your blood pressure and pulse rate will be taken twice during weekly visits, two minute apart from each measurement after five minutes of rest. Finally, your body height will be measured during visit 3 and the circumference of your waist and hip will be taken at each study visit.

**Blood Collection:** During each study phase your blood will be taken at each study visit. From these samples, we will monitor your health and store the remainder for future use if appropriate. These blood collections will help us measure blood substances (like nutrients, hormones, proteins and fats) in your body during the study.

The total amount of blood to be collected from you is approximately 300mls (20 tablespoons). This is less than the Red Cross standard amount for blood donation of 500ml in 56 days.

**Questionnaires:** We will monitor health status, medication use, lifestyle habit, dietary intake, physical activity and functional status though several questionnaires that will be given to you through the study.

**Scheduling Conflicts:** If you become sick or have a scheduling conflict, you may be unable to complete a 1-week diet phase or return as scheduled from your break between

the diet phases. An effort will be made (if you choose to continue) to extend the study diet, reschedule your start day, or restart the diet phase. Study tests and procedures may be rescheduled within the new time frame.

**Fasting before visits:**

12-hour fasting is required before visits 3-8 (water will be OK). If medication is needed it can be taken with breakfast at the HNRCA.

# RISKS

There is a potential risk for allergic reactions to food ingredients in the study diets. In order to minimize this risk an MRU Dietician conducts a diet history, which includes assessment for food allergies and intolerances at screening. Subjects with a history of allergies to food ingredients in the study diets will not be allowed to participate in this study.

All blood draws are performed by the MRU nursing staff. The risk associated with blood collections may include minor, temporary discomfort or pain associated with the needle stick itself (venipuncture), bruising, infection or inflammation of the vein (phlebitis).

You may feel hungry after you have had nothing to eat/drink (except water) for the previous 12 hours before study visits.

All procedures will be done by experienced study staff to minimize any discomfort that you may experience.

There may be a small risk to your confidentiality; however these risks have been minimized to the extent possible.

If revealed, the results of genetic testing could possibly affect your employment or your ability to purchase health and/or life insurance. However, Federal law “the Genetic Information Nondiscrimination Act” protects you from such discrimination in employment and health insurance. These protections do not extend to workers in companies with fewer than 15 employees. The results of any genetic testing involving your saliva will be kept in strictest confidence and will not be entered into your medical record.

You will be informed of any significant new findings developed during the course of this research, which may relate to your willingness to continue participation.

# BENEFITS

There will be no direct benefit to you from being in this study.

# ALTERNATIVES

Your alternative is to not participate in the research study.

# RESEARCH RELATED INJURY

Emergency medical treatment will be given to you if you are hurt or get sick as a direct result of being in this research study. You or your insurance carrier will be required to pay for any such medical care. Any needed medical care is available at the usual cost. All needed facilities, emergency treatment, and professional services are available to you, just as they are to the general public. The institution will not pay for your treatment if you become ill or injured as part of this study.

# COSTS

There are no costs to you associated with your participation in this research study.

# PAYMENT

You will receive a stipend payment of $460.00 for completing the entire study (all 8 study visits (including both screening visits) paid in installments as follows: $15.00 upon completion of Screening 1; $25.00 upon completion of Screening 2; $120.00 upon completion of Diet Phase 1 and $300.00 upon completion of Diet phase 2.

If you withdraw from the study before completing all study visits, you will be paid in proportion to the number of visits you completed as follows:

| **Pro-rated payment if early withdrawal** | | |
| --- | --- | --- |
| **Payment per visit** | | **Total Payment** |
| Visit 1 (screening 1) | $15 | Completed Visit 1 = $15 |
| Visit 2 (screening 2) | $25 | Completed Visits 2: 1 & 2 = $40 |
| Visit 3 (intervention 1) | $40 | Completed Visits: 1, 2 & 3 = $80 |
| Visit 4 (intervention 2) | $40 | Completed Visits: 1, 2, 3 & 4 = $120 |
| Visit 5 (intervention 3) | $40 | Completed Visits: 1, 2, 3,4 & 5 =$160 |
| Visit 6 (intervention 4) | $40 | Completed Visits: 1, 2, 3, 4, 5 & 6= $200 |
| Visit 7 (intervention 5) | $80 | Completed Visits: 1, 2, 3, 4, 5, 6 & 7 = $280 |

The payments will be mailed to you as a check after completion of the study. You should expect to receive the check within 2-3 weeks after mailing. However, in the event of government shutdown or other emergency situations, payments will be delayed until the shutdown/emergency ends and all systems are restored at the HNRCA.

Due to federal tax law, you are required to provide us your social security number in order to process your payments. If you receive over $600 from Tufts University Health Sciences in a single calendar year (either in a single study or multiple studies), you will be issued an IRS 1099 form. This may affect your taxes. Only payments for being in

research studies will be used to decide if you should receive the IRS form. Money for study-related parking, food and other expenses are not included in this IRS disclosure.

If employee-subjects qualify to participate in the study, they cannot participate as volunteers during hours in which they are being compensated by Tufts University for their regular work. You cannot use vacation, personal days and sick time to participate in the study.

# PRIVACY AND CONFIDENTIALITY

The records identifying your name will be kept confidential and, to the extent permitted by the applicable laws and/or regulations, will not be made publicly available. The results of the study will only be published or presented as group data. No individual participants will be identified. Data forms will be identified with a unique study number and kept locked in the study office.

The blood sample you provide will be labeled with unique identifiers and transferred to the Genomics and Nutrition Laboratory and Nutrition Evaluation at the HNRCA.

If you agree to take part in this research study, your personal information will not be given to anyone unless we receive your permission in writing. It will only be given if the law requires it. It will also only be given for regular hospital treatment, payment, and hospital management activities.

We will make every effort to keep your information private, but it cannot be completely guaranteed. Certain government agencies (Office for Human Research Protections, Department of Health and Human Services, Food and Drug Administration) and the Institutional Review Board of Tufts Medical Center and Tufts University Health Sciences

may check records that identify you. This might include your medical or research records and the informed consent form you signed. The records of this study might also be reviewed to make sure all rules and guidelines were followed. Your medical and research records will be kept on file for a period of at least 7 years.

A description of this clinical trial will be available on http://www.Clinical Trials.gov, as required by US Law. This Web site will not include information that can identify you. At most, the Web site will include a summary of the results. You can search this web site at any time.

# WHOM TO CONTACT

Should you have any problems or questions about this research study and our screening procedures, you may contact Dr. Jose Ordovas, the study Principal Investigator, at:

Dr. Jose Ordovas HNRCA

711 Washington Street

Boston, MA 02111

You could also call Dr. Ordovas at his office (617) 556-3102 during daytime (9am to 5pm), Cell: (508)904-5455 or email him at [jose.ordovas@tufts.edu](mailto:jose.ordovas@tufts.edu)

You may also call other members of the research team:

Lucia Pham BSc: (617) 556-3245, days

Lisa Ceglia MD (617) 556- 3085, days

For emergencies at any time, including days, evenings, weekends and holidays, please call (617) 230-7545 to reach Dr. Ceglia, the study physician.

**Documentation of Consent**

I have been given a copy of this form. I have read it or it has been read to me. I understand the information and have had my questions answered to my satisfaction. I agree to take part in this study.

I understand that I will be informed of any new findings developed during the course of this research study that may affect my willingness to stay in this research study.

Date Participant’s Signature

I have fully explained to the nature and purpose of the above-described study and the risks that are involved in its performance. I have answered all questions to the best of my ability.

Date Principal Investigator or Representative’s Signature
